# Fast multi-core based multimodal registration of 2D cross-sections and 3D datasets

**DOI:** 10.1186/1471-2105-11-20

**Published:** 2010-01-11

**Authors:** Michael Scharfe, Rainer Pielot, Falk Schreiber

**Affiliations:** 1 Leibniz-Institute of Plant Genetics and Crop Plant Research (IPK), Corrensstr. 3, D-06466 Gatersleben, Germany; 2 Leibniz-Institute for Neurobiology (IfN), Brenneckestr. 6, D-39118 Magdeburg, Germany; 3 Institute of Computer Science, Martin Luther University Halle-Wittenberg, Von-Seckendorff-Platz 1, D-06120 Halle, Germany

## Abstract

**Background:**

Solving bioinformatics tasks often requires extensive computational power. Recent trends in processor architecture combine multiple cores into a single chip to improve overall performance. The Cell Broadband Engine (CBE), a heterogeneous multi-core processor, provides power-efficient and cost-effective high-performance computing. One application area is image analysis and visualisation, in particular registration of 2D cross-sections into 3D image datasets. Such techniques can be used to put different image modalities into spatial correspondence, for example, 2D images of histological cuts into morphological 3D frameworks.

**Results:**

We evaluate the CBE-driven PlayStation 3 as a high performance, cost-effective computing platform by adapting a multimodal alignment procedure to several characteristic hardware properties. The optimisations are based on partitioning, vectorisation, branch reducing and loop unrolling techniques with special attention to 32-bit multiplies and limited local storage on the computing units. We show how a typical image analysis and visualisation problem, the multimodal registration of 2D cross-sections and 3D datasets, benefits from the multi-core based implementation of the alignment algorithm. We discuss several CBE-based optimisation methods and compare our results to standard solutions. More information and the source code are available from http://cbe.ipk-gatersleben.de.

**Conclusions:**

The results demonstrate that the CBE processor in a PlayStation 3 accelerates computational intensive multimodal registration, which is of great importance in biological/medical image processing. The PlayStation 3 as a low cost CBE-based platform offers an efficient option to conventional hardware to solve computational problems in image processing and bioinformatics.

## Background

**Figure 1:**
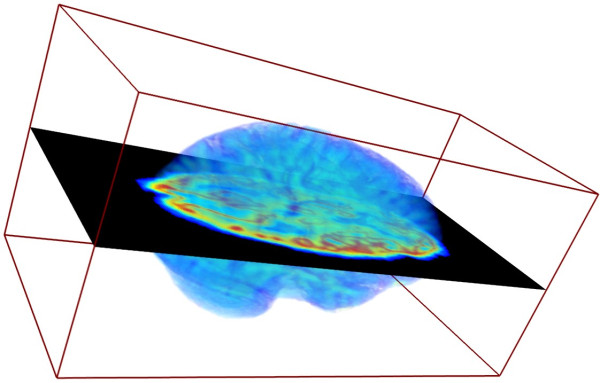
**A volume rendering of the 3D NMR dataset of a brain (see Figure 9) together with a registered 2D PET scan (see Figure 10) after the multimodal alignment procedure**.

Comprehensive understanding of biological structures requires sophisticated techniques in many areas such as the combination of 2D and 3D images or models of biological objects. Examples are the integration of histological cross-sections providing structural information and development-specific distribution patterns of mRNA, metabolite concentrations or enzyme activities into a 3D morphological framework [1], the combination of 2D computer tomography (CT) slices with a 3D atlas [2], or the integration of 2D positron emission tomography (PET) slices, providing information about metabolic activity, into a 3D NMR dataset, see Figure 1. For reconstruction and 3D visualisation these 2D cross-sections have to be registered at correct spatial positions in a 3D morphological framework. Manual registration of cross-sections is tedious, subjective and very time-consuming. The accurate registration of images, obtained by diverse imaging techniques, requires automatic multimodal alignment techniques, which is an important research field in biological and medical image processing [3-5]. Such approaches determine the optimal spatial position on the base of suitable similarity functions, such as cross correlation [6] or mutual information [3,7,8]. Registration by automatic procedures still requires extensive computational resources, so that fast algorithms and the implementation on parallel hardware would greatly enhance the feasibility of these investigations. The multi-core Cell Broadband Engine (CBE) allows fast parallel computation on eight cores per chip, presenting potential as a target for implementation of image registration algorithms [9,10]. In this paper we implemented and evaluated a multimodal alignment approach based on mutual information on the CBE. The availability of the inexpensive CBE-driven PlayStation 3 provides the opportunity to simultaneously align a high number of image stacks on a low-cost platform and therefore improves the automatic analysis and visualisation of biological information obtained through diverse imaging methods. We discuss several CBE-based optimisation methods and compare our results to standard solutions.

### Cell Broadband Engine

**Figure 2:**
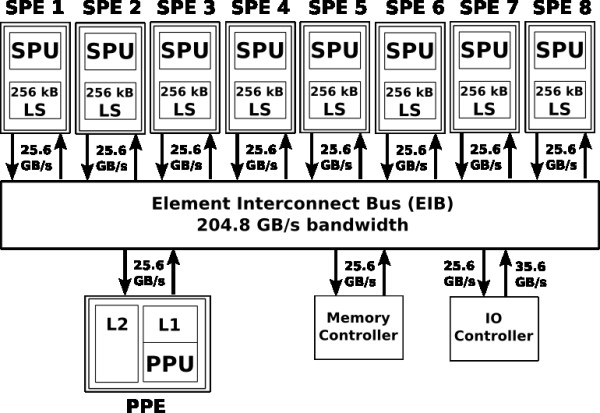
**Architecture of the Cell Broadband Engine**. Eight Synergistic Processing Elements (SPEs) perform up to 210 GFLOPs. One 64-bit Power Processing Element (PPE) manages the task scheduling. LS denotes the local storage of one SPE.

The Cell Broadband Engine is a microprocessor architecture developed by Sony Computer Entertainment, Toshiba and IBM to provide power-efficient and cost-effiective high-performance processing for a wide range of applications. The first-generation Cell processor combines a Power Processor Element (PPE) with eight Synergistic Processor Elements (SPEs) [11]. The PPE contains a 64-bit PowerPC Architecture core (PPU) and can run 32- and 64-bit operating systems and applications. Each SPE contains a RISC core (SPU) which is optimised for computational intensive Single-Instruction-Multiple-Data (SIMD) applications. A single SPE can perform up to eight single precision (SP) operations per cycle so that all SPEs provide a theoretical peak performance of about 210 GFLOPS. All nine computational units communicate with each other, the main memory and I/O devices through the Element Interconnect Bus (EIB), which provides a bandwidth of 25.6 GByte to each component and a total bandwidth of 204.8 GByte/sec [12]. Figure 2 shows an overview of the initial implementation of Cell Broadband Engine.

There are various types of Cell-based systems available, for example, IBM offers blades with two Cell processors and several GByte of RAM, appropriate for high performance cluster computing. Sony released the PlayStation 3 game console, equipped with a low cost version of the Cell processor. This version contains seven operating SPEs (only six of them are available for applications) and only 256 MB RAM [13]. However its price (about 300 Euro) makes it attractive as an alternative high performance platform.

## Methods

This section is organised as follows: first we describe the pre-processing of typical 2D and 3D image datasets and then we give a brief description of the automatic multimodal alignment procedure. The last subsection describes the implementation and optimisation of the algorithms to the CBE in detail.

### Image Processing

The task of multimodal alignment is to register 2D images into a 3D image dataset. The 2D dataset is given as (, ) with 0 ≤ *i *< and 0 ≤ *j *< and the 3D dataset is given as as (, , ) with 0 ≤ *i *<, 0 ≤ *j *< and 0 ≤ *k *< with the same resolution as the 2D dataset. If necessary, the images have to be adjusted to the same resolution by a pre-processing step. The 2D dataset could be, for example, a cross-section cut, a 2D CT or a 2D PET slice; the 3D dataset could be, for example, a NMR dataset, a CT dataset or a 3D atlas.

Multimodal alignment is a typical image analysis problem. For the 2D/3D alignment presented here we assume that the direction at which the 2D image should be aligned is given, for example, by the experimental procedure. Without loss of generality, this direction is the *z*-direction of the 3D dataset. However, if the direction is not given the algorithm could be easily extended to also find the correct direction, resulting in a heavily increased computing time.

The 2D/3D alignment procedure is divided into successive 2D/2D alignments. A similarity function (e. g. cross correlation [6] or mutual information [3,7,8]) determines for each slice *k *of the 3D dataset in *z *direction the optimal translation parameters in *x *and *y *direction and the optimal rotation-angle in the *xy*-plane of the image (*x*^2*D*^, *y*^2*D*^). To determine the best parameters, all possible combinations of parameters within the search space were used for the calculation. The highest similarity value depicts the optimal registration. To reduce the effort, often the search space is restricted by prior knowledge. In this study, we used a similarity function based on normalised mutual information, which is very suitable for registration of multimodal registration [3,4]. Given two probability distributions *p*_*T *_(*t*), *p*_*F *_(*f*) and the joint probability *p*_*TF *_(*t, f*) of target image *T *and floating image *F*, the normalised mutual information *NMI*(*T, F*) is defined by means of the Kullback-Leibler measure [14]:(1)

### Multimodal alignment procedure

**Figure 3:**
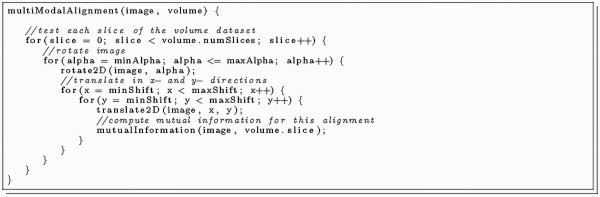
**Listing 1: The sequential multimodal alignment procedure**.

The sequential alignments of the 2D slices require a high amount of computing time, because each alignment is independent from another and each parameter combination has to be calculated. Program listing 1 (Figure 3) shows the main routine of a sequential multimodal alignment implementation. The execution time of each subroutine scales well with the size of the dataset so that the majority of the runtime is spent on the inner loop, respectively on the translate- and mutual-information procedures. Optimisations on both these code sections promise the best computation time speedups.

### Implementation on the Cell Broadband Engine

To exploit the considerable performance of the CBE, architecture-specific properties have to be considered. Well-known sequential programs have to be re-designed and parallel concepts and new architecture-specific restrictions have to be taken into account. Using these features it is possible to obtain optimisation results close to the peak performance of the processor [15,16]. In the case of our implementation we achieved significant improvements by following these rules:

1. Schedule the tasks onto all cores (partitioning)

2. Avoid scalars and use vectors instead (vectorisation)

3. Eliminate and reduce branches on the SPE-code (branch reduction)

4. Avoid 32-bit Integer multiplies on the SPEs (avoiding Int32 multiplications)

5. Manually unroll loops on the SPE-code (unrolling)

6. Pay attention to the limited local storage of the SPE (limited local storage)

Our algorithm consists of a multi-threaded alignment procedure with one thread for each available SPE for the computing work and one manager thread on the PPE managing data-transfers, task-scheduling and I/O operations. The application source code was implemented in C with SIMD extensions and SPE intrinsics provided by IBM's Software Development Kit (SDK) for multi-core Acceleration [17-19].

#### 1) Partitioning

The design of a parallel algorithm often requires an efficient partitioning of the computations between the available processing units. In the case of the CBE it is recommended that the SPEs performs all heavy computational tasks and the PPE acts as a control unit to organise the task flow, I/O and data transfer operations [20]. The first step in optimising the sequential multimodal alignment program was to break the tasks into discrete portions of work that can be distributed to all available SPEs. Due to the iterative structure of the algorithm, the 3D dataset can be easily decomposed such that each parallel task works on a portion (slice) of the data.

**Figure 4:**
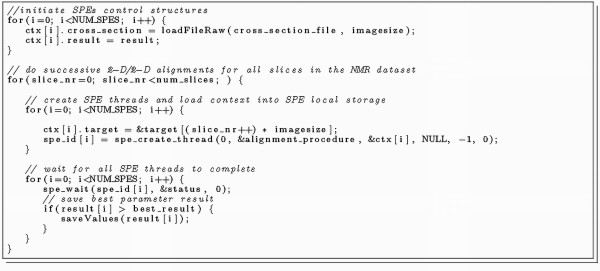
**Listing 2: The program running on the PPE manages the task scheduling (NUM_SPES denotes the number of available SPEs on the Cell Processor) procedure**.

The PPE organises a job queue to process a fixed amount of independent jobs and sends each SPE one slice of the 3D dataset while there is still a slice left to align. Program listing 2 (Figure 4) shows a code fragment of the program running on the PPE which manages the task scheduling onto the SPEs. In order to fully exploit the available power of the Cell processor, the PPE should also be involved in the calculations. This requires additional programming effort because the SPEs are much faster in processing than the PPE and they should be supplied immediately with new tasks to reduce unnecessary idling. Due to the excellent predictable performance of the SPEs (on branchless code) it is possible to stop the PPE calculations at a certain time and manage the job queue without major delays.

#### 2) Vectorisation

**Figure 5:**
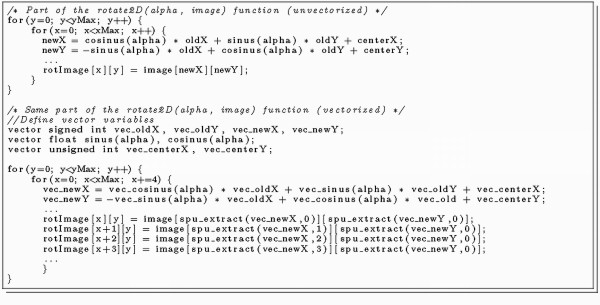
**Listing 3: This code fragment of the image rotate function shows some elementary changes from standard to vectorised instructions**.

The main part of the Cell Processors performance lies in its SPEs, which are SIMD vector processors. They achieve high performance by using large register files (128 × 128 bit) and significant speedup can be achieved using SIMDisation (vectorisation). For any given algorithm, vectorisation can usually be applied in different ways. Sometimes it is simple and intuitive to aggregate a set of variables into a vector and perform one operation on it instead of successive operations on each variable. Scalars, which are not appropriate for vectorisation, should be converted to quad-word vectors to avoid wasted instructions for loading and storing them [15]. Some compilers do auto-vectorisation, but their capabilities remain limited, so it is recommended to do this task manually. IBMs Cell SDK provides several useful C/C++ language extensions, mainly vector data types and operations on these data types [18]. We applied manual vectorisation to all time critical functions to achieve a higher overall performance on the Cell SPEs. Program listing 3 (Figure 5) illustrates such a code modification using IBMs SDK C language extensions [18].

#### 3) Branch reduction

**Figure 6:**
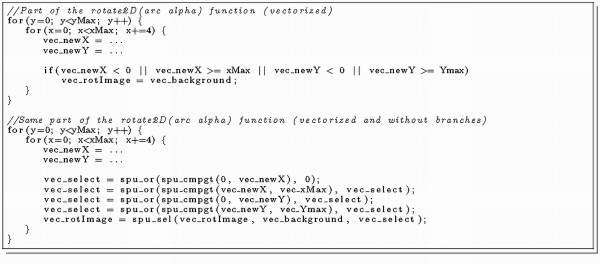
**Listing 4: Example of how to eliminate branches with IBM SDK instructions**.

The SPEs do not provide dynamical branch prediction and a mis-predicted branch leads up to 19 wait cycles [16]. To avoid this, static hint branch instructions can be used to indicate the fetch direction or the source code can be made branchless by computing all possible results and selecting the correct one [15]. Program listing 4 (Figure 6) shows an example of how to eliminate an expensive if-else condition. This optimisation resulted in more code lines and more single calculations, but requires much less computation time on a SPE. Therefore, variable execution times due to mis-predicted branches were eliminated, leading to very predictable SPE calculation times.

#### 4) Avoidance of int32 multiplications

Because the current SPE contains only a 16 × 16 bit multiplier, 32-bit integer multiplies requires four extra instructions [16]. Therefore *unsigned shorts *should be used if possible and arrays should have power-of-two size to avoid multiplication when indexing.

#### 5) Unrolling

**Figure 7:**
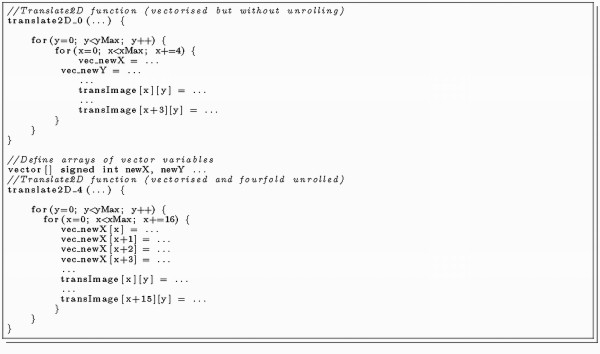
**Listing 5: This code fragment illustrates a fourfold unrolling of a typical nested loop**.

The technique of loop unrolling provides significant performance improvements, as compilers can automatically schedule operations and optimise computations, if the algorithm consist of many independent operations [15,21]. In particular nested loops have been unrolled manually to gain a considerably better performance. It seems to be useful to try several levels of unrolling in order to find an optimal usage of the SPE's large register file. An example of a fourfold unrolled nested loop is shown in program listing 5 (Figure 7).

#### 6) Limited local storage of the SPE

Each Synergistic Processor Element (SPE) has its own 256 KByte RAM for instructions and data which is called local storage (LS) [11]. The SPEs can only execute code in the LS and only operate on data residing in this storage. Instead of direct main memory access, the SPE has a programmable DMA controller which performs transfers between main memory and LS [12].

Our goal for the high-performance implementation of multimodal alignment was to keep all memory requirements of a SPE thread in the LS. The size of our SPE program is 58 KByte. In our application examples (see Results section) each 2D-image and each 3D-slice is a 256 × 175 8-bit gray-value pixel image, thus we need about 90 KByte for storing the data. The approximately 108 KByte left on the LS are sufficient to store intermediate results and temporary variables. The advantage of this approach is that no additional data transfer is necessary.

## Results

### Evaluation Platforms

The algorithms were implemented in C with special extensions for vector and SIMD purposes provided in IBMs CBE SDK 3.0 [17-19]. For performance tests we used a first-generation stand-alone PS3 as an inexpensive Cell BE platform [22]. Yellow Dog Linux 6.1 with kernel 2.6.23-9 was installed on the console and the source-code was compiled with the GNU c compiler (gcc) version 4.1.1. The programs can be found as Supplementary Material Additional file 1.

**Figure 8:**
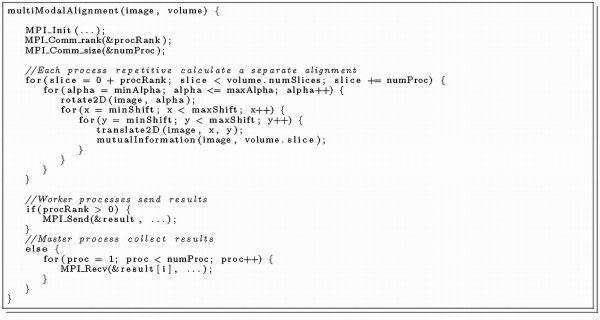
**Listing 6: The MPI parallelised multimodal alignment procedure**.

We compared the performance of our CBE-optimised alignment program to a *Message Passing Interface *(MPI) parallelised version on a common quad-core Opteron system [23]. Similar to the described partitioning optimisation for the CBE, the task was divided amongst all processor cores. Not surprisingly the performance scaled well with the number of used cores. Program listing 6 (Figure 8) shows the main routine of the MPI-parallelised multimodal alignment procedure. We tested this implementation on a workstation equipped with two AMD Opteron 2356 (2.3 GHz), 16 GByte RAM and an Open Suse Linux 11.0 with kernel 2.6.25.11. On this platform we compiled the source-code with gcc 4.3.1 (optimisation level 5) and tested it with OpenMPI 1.2.5 and different amounts of parallel used cores.

### Evaluation Example

**Figure 9:**
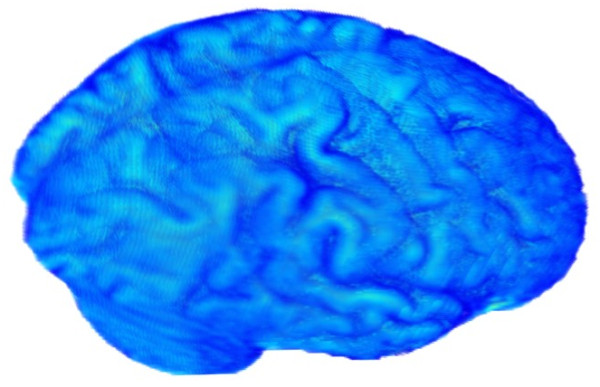
**A volume rendering of a NMR dataset of a brain**.

**Figure 10:**
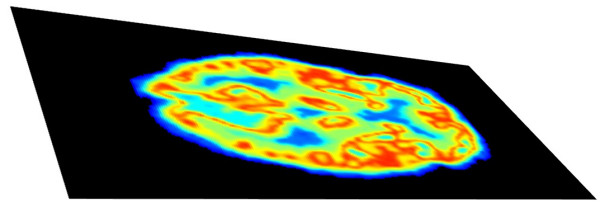
**A 2D PET image of a brain**.

**Figure 11:**
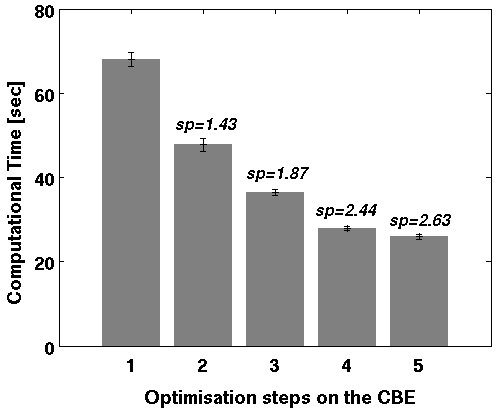
**Mean computation time of one single alignment (including standard deviation) and speedup (sp) after each optimising step: (1) partitioning, (2) vectorisation, (3) reduce branches, (4) avoid Int32 multiplies, (5) explicit unroll**. Speedup (sp) compares the optimised solution to a simple partitioning (1) on the CBE and shows the effect of each optimisation step.

**Figure 12:**
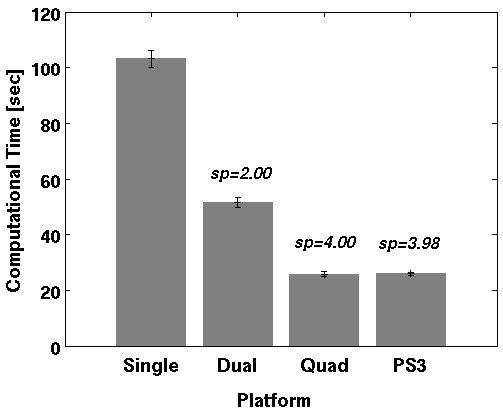
**Comparison between the mean computation times of one single alignment on the PS3 Cell Processor and a MPI-parallelised solution on an Opteron performed on the example datasets**. Speedup (sp) compares each platform solution to the single-core solution on the Opteron. The average speedup of the PS3 Cell is 3.98 compared to the single-core Opteron, 1.99 compared to dual-core Opteron and 0.99 to quad-core Opteron solution.

**Figure 13:**
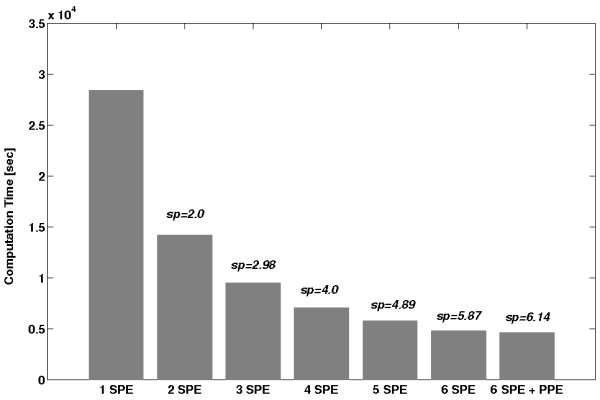
**Computational time of a typical multimodal alignment task depending on the number of CBE components used**. Speedup (sp) compares each solution to the single SPE solution. The runtime scales well with the number of utilised SPEs. The additional usage of the PPE resulted in only minor speedups.

In this study, we used two 3D NMR datasets of the male and female brain, freely available from the Open Access Series of Imaging Studies (OASIS) project [24]. The dimensions of the 3D images were 256 × 175 × 176 voxel, an example of the data is shown in Figure 9. Three modified slices of each NMR datasets and three different 2D PET scans (see Figure 10 for an example), published by the National Institute of Aging [25], were used for registrations on the brain data. The 2D images were converted into gray-values and down-scaled to the respective resolution of the 3D dataset. Because of a given rough pre-alignment the search space could be constrained for the translation from -30 to +30 pixel and for the rotation-angle from -20° to +20°. Figure 1 shows an example of the multimodal registration of a 3D dataset (brain) and an associated 2D image (PET). The results of the analysis are detailed below and shown in Figures 11, 12 and 13.

### Optimisation Results on the PS3

To realise the optimisation steps described above and access the high performance features of the CBE processor, we used a set of arithmetic, compare, logical scalar and mask intrinsics [18,20]. A timer measured the period of the time-critical calculations in the alignment procedure. The differences between the results for each optimisation-step (see section Methods) was an indicator for its effectiveness. We repeated each benchmark-test several times with different combinations of the 3D and 2D images and compared the means of their computation time with each other.

#### 1) Partitioning

As a first step we distributed the calculations on all available processor cores (decomposition). At the beginning of the calculations, the PPE loaded the 3D volume and the 2D-image, created one thread for each SPE and transferred via DMA the 2D-image and disjunct NMR-slices to the SPEs. After receiving them, the SPEs computed their local alignment and returned the alignment-parameters to the PPE which stored the best of these alignments. This was repeated with the next layers of the volume until all slices had been processed. Not surprisingly, the execution time of the whole alignment scales well with the number of used SPEs (see Figure 13). Because the sum of all transfer times took only a small fraction of the overall execution time, overlapped techniques such as double buffering were not implemented.

Partitioned alignment, without further optimisations, required an average computation time of 67 seconds per NMR slice. This is an average speedup of 1.49 compared to a single-core Opteron solution, but it does not exhaust the whole potential of the CBE processor.

#### 2) Vectorisation

The SPEs vector architecture requires vectorised source-code to achieve high performance [15,16]. SPEs then have the ability to compute similar operations on several variables in each cycle. We extensively transformed single variable operations to vector variable operations. Because of the recurring dataflow in the main computational routines (see Methods/Multimodal alignment procedure) this was applicable in a straightforward manner. The speedup of 1.43 gained from this optimisation was surprisingly not an outstanding result but may relate to the powerful auto-vectorisation support of the Gnu C compiler [26]. However, manually implemented vectorisation provided a significant speed enhancement whereby the PlayStation 3 achieved an acceptable performance in comparison with modern standard processors. In the case of our implementation, partitioning and vectorisation provides a speedup of 2.12 compared to a single-core Opteron, thus reaching the speed of a dual-core Opteron version parallelised with MPI.

#### 3) Reduce branches and avoid Int32 multiplications

As described in the Method section, we implemented branchless code and reduced 32-bit Integer multiplies as far as possible. Because the multimodal alignment functions contain many conditions (branches), this technique raised the performance significantly. Branchless code with less Int32 multiplications resulted in a speedup of 3.65 compared to a single-core Opteron solution.

#### 4) Explicit unroll

As a last optimisation step, we explicitly unrolled loops to benefit from the large register (128 × 128 bit) on each SPE. The used GNU C compiler offers automatic loop unrolling mainly on simple loops (not nested and without dependencies), so in many cases a manual unrolling can result in considerable performance improvements. In our evaluation example, two- and four-times unrolling led to only minor speedups. A possible explanation besides existing compiler optimisations is that in most cases the SPEs registers were nearly completely filled by the assigned data in one single loop cycle; therefore no further significant speedup could be achieved by additional unrolling.

The tests using all optimisation steps show an average speedup of 3.97 compared to a single-core Opteron for the registration of a 2D PET scan. Figure 11 shows the benchmark results after each optimisation step with corresponding speedups.

It should be mentioned that the PPE also calculated alignments on some slices. However, this reduced the overall execution only slightly. We also investigated the performance of the PPE in comparison to one SPE. Our tests show a speed advantage by a factor of four of the optimised SPE source-code compared to a vectorised PPE version. A performance comparison of the optimised CBE alignment program to the MPI-parallelised version is shown in Figure 12. The CBE program is nearly (99%) as fast as the MPI-parallelised program computed on four Opteron cores. Due to the strict data parallelism of our task a single core Opteron reached only about a quarter and a dual core about a half of this performance. This corresponds to an average speedup of 3.97 of the optimised CBE alignment compared to the single-core Opteron and of 1.98 to the dual-core Opteron, respectively. Ohara *et al*. [9] reported a similar approach, where they implemented a mutual information based linear registration of monomodal 3D MRI images. The speedup factors in their study are lower (5.8 on 16 SPEs compared to a 3,0 GHz Woodcrest Intel Xeon (one core)), but a direct comparison with our results (3.97 on 6 SPEs compared to a 2,3 GHz Opteron 2356 (one core)) is difficult. In addition, their registration algorithm is based on Matte's mutual information approach as implemented in Insight Imaging Toolkit (ITK) [27] library. However, this fast multi-resolution algorithm does not work well with specific NMR data such as NMR data of barley seeds which we are currently investigating.

## Discussion and Conclusion

In this paper, we have presented a set of optimisation steps to accelerate the computation of a multimodal alignment, a typical image analysis problem, on the Cell Broadband Engine in a PlayStation 3. This platform seems to be an attractive solution for high performance computing due its considerable high peak performance and its low cost (about 300 Euro). An optimised CBE application is very predictable in its execution time and with the knowledge of architecture-specific properties it is possible to reach nearly the peak performance of this processor. The bottleneck in this algorithm is the computation of the NMI function, which requires most of the computing time. There is only low communication as for typical image sizes (as in our examples) the program and data fit into the local storage area of the SPEs. Potential further developments would be the investigation of DMA transfer effects for images of bigger size and comparison with other platforms such as graphics processing units.

Developing efficient code for the CBE requires several optimisation techniques. Furthermore, the optimised source-code is not easily portable to other architectures. Nevertheless, the comparison with the average execution times on an Opteron system shows that in case of our application the CBE processor in the PlayStation 3 (with only six SPEs) achieves an average speedup of 3.97 compared to a single-core Opteron. It requires at least four physical Opteron cores to reach the speed of the console. Considering the price of the quad-core AMD processor (about 600 Euro) included in a basic workstation (about 1000 Euro), the PS3 will meet their reputation as a low-cost high-performance computing platform. Therefore the applicability of the Cell Broadband Engine for common problems in bioinformatics is of current interest and several approaches have been presented [28-30]. We believe that this platform is an interesting alternative for fast multimodal alignments of 2D and 3D datasets and is able to speedup other tasks in image processing.

## Authors' contributions

RP and FS designed the study, MS implemented the method, MS, RP and FS analysed the results and wrote the paper, all authors read and approved the final manuscript.

## Supplementary Material

Additional file 1 Yellow Dog Linux 6.1 with kernel 2.6.23-9 was installed on the console and the source-code was compiled with the GNU c compiler (gcc) version 4.1.1. Click here for file
